# QTL mapping reveals genetic determinants of fungal disease resistance in the wild lentil species *Lens ervoides*

**DOI:** 10.1038/s41598-017-03463-9

**Published:** 2017-06-12

**Authors:** Vijai Bhadauria, Larissa Ramsay, Kirstin E. Bett, Sabine Banniza

**Affiliations:** 1 Crop Development Centre/Department of Plant Sciences, University of Saskatchewan, Saskatoon, Canada; 2 Swift Current Research and Development Center, Agriculture and Agri-Food Canada, Swift Current, Canada

## Abstract

*Lens ervoides*, a wild relative of lentil is an important source of allelic diversity for enhancing the genetic resistance of the cultivated species against economically important fungal diseases, such as anthracnose and Stemphylium blight caused by *Colletotrichum lentis* and *Stemphylium botryosum*, respectively. To unravel the genetic control underlying resistance to these fungal diseases, a recombinant inbred line (RIL) population (n = 94, F_9_) originating from a cross between two *L. ervoides* accessions, L01-827A and IG 72815, was genotyped on the Illumina HiSeq 2500 platform. A total of 289.07 million 100 bp paired-end reads were generated, giving an average 7.53-fold genomic coverage to the RILs and identifying 2,180 high-quality SNPs that assembled in 543 unique haplotypes. Seven linkage groups were resolved among haplotypes, equal to the haploid chromosome number in *L. ervoides*. The genetic map spanned a cumulative distance of 740.94 cM. Composite interval mapping revealed five QTLs with a significant association with resistance to *C. lentis* race 0, six QTLs for *C. lentis* race 1 resistance, and three QTLs for *S. botryosum* resistance. Taken together, the data obtained in the study reveal that the expression of resistance to fungal diseases in *L. ervoides* is a result of rearrangement of resistant alleles contributed by both parental accessions.

## Introduction

Lentil (*Lens culinaris* Medik. ssp. *culinaris*) is the world’s fifth largest pulse crop with annual production of 4.89 Mt^1^. It is an annual self-pollinating, diploid (2*n* = 2*x* = 14), cool season legume, with a relatively large genome (~4 Gb)^2^. The domestication of lentil dates back to 8000 BC in the Fertile Crescent^3^, and the crop is curently produced in over 70 countries around the globe with Canada, India, Australia, Turkey and Nepal being the top five producers^1^. High in protein, fiber, micronutrients and vitamins, lentil provides nutritional security on a global scale^4^.

A recurring challenge to lentil production is the significant seed yield loss and down-grading of seed quality due to fungal diseases. Anthracnose, caused by *Colletotrichum lentis* Damm, and Ascochyta blight, due to infection by *Ascochyta lentis* Vassilievsky, are major diseases of lentil and account for 70–78% crop loss under high disease pressure in Canada^5–8^. Stemphylium blight, caused by *Stemphylium botryosum* Wallr., causes signicant seed yield loss in Noth-East India, Nepal and Bangladesh^9, 10^ and is an emerging disease in Canada^11^.

Wild relatives of crops can serve as a source of allelic diversity for disease resistance, presumably due to an ongoing co-evolutionary arms race between pathogens and their crop hosts wherein the pathogens tend to evolve their effectors at a higher rate to dismantle the microbe-associated molecular pattern and effector-triggered immunity in the plant, leading to resistance erosion^12^. Seven taxa have been described in the genus *Lens*: the domesticated *L. culinaris* ssp. *culinaris* and its wild relatives *L. culinaris* ssp. *orientalis* (Boiss.) Ponert and *L. tomentosus* Ladiz. in the primary genepool, *L. odemensis* Ladiz. and *L. lamottei* Czefr. in the secondary genepool, *L ervoides* (Brign.) Grande in the tertiary genepool and *L. nigricans* (Bieb.) Godr. in the quaternary genepool^13^. The wild species are distributed along the Mediterranean basin and further East into Central Asia^14^. Germplasm screening of wild species for resistance to several pathogens led to the identification of the two *L. ervoides* accessions, L01–827A and IG 72815, with superior resistance to anthracnose, Ascochyta blight and Stemphylium blight^15–19^. Resistance to *A. lentis* and *C. lentis* race 1, as well as to *C. lentis* race 0, for which no high level of resistance has been found in cultivated lentil, was transferred from *L. ervoides* accession L01-827A to cultivated lentil through crosses with Eston and CDC Redberry using interspecific hybridization and embryo rescue^16, 18^. Mapping of quantitative trait loci (QTLs) controlling resistance has been impeded by segregation distortion in interspecific populations, so selection to date has relied on conventional phenotyping after challenge with the pathogens.

Recent advances in next-generation sequencing chemistry and platforms have accelerated the discovery of high-density molecular markers, especially single nucleotide polymorphism (SNP) markers, although the resolution of QTLs remains challenging for those crops with complex and large genomes, such as lentil. To address this limitation, several genome complexity reduction-based genotyping approaches have been developed and used in the discovery of high-density SNP markers. Such approaches use restriction enzymes to reduce genome representation and multiplexing prior to high-throughput sequencing^20, 21^.

In this study, we genotyped an intraspecific biparental recombinant inbred line (RIL) population (F_9_), originating from a cross between *L. ervoides* accessions L01-827A and IG 72815, on the Illumina HiSeq 2500 platform using *Pst*I-*Msp*I- based genotyping-by-sequencing (GBS) with the objective to generate a high-density genetic linkage map suitable for QTL analysis. A total of 289,072,291 100-bp paired-end reads across 94 RILs were generated, providing an average 7.53-fold genomic coverage to RILs with 3,011,170 reads per RIL. A total of 2,180 high-quality SNP variants were called from the reads that were uniquely mapped onto the *L. culinaris* cv. CDC Redberry genome assembly^22^ and assembled in 543 unique haplotypes consisting of 368 genetic bins and 175 singletons. Seven linkage groups were resolved among haplotypes equal to the haploid chromosome number in the genus *Lens*. Furthermore, QTLs controlling resistance *to C. lentis* races 0 and 1, and *S. botryosum* were mapped onto the genetic linkage map.

## Results

### The L01-827A × IG 72815 RIL population segregates for resistance to *C. lentis* races 0 and 1, and *S. botryosum*

**Figure 1:**
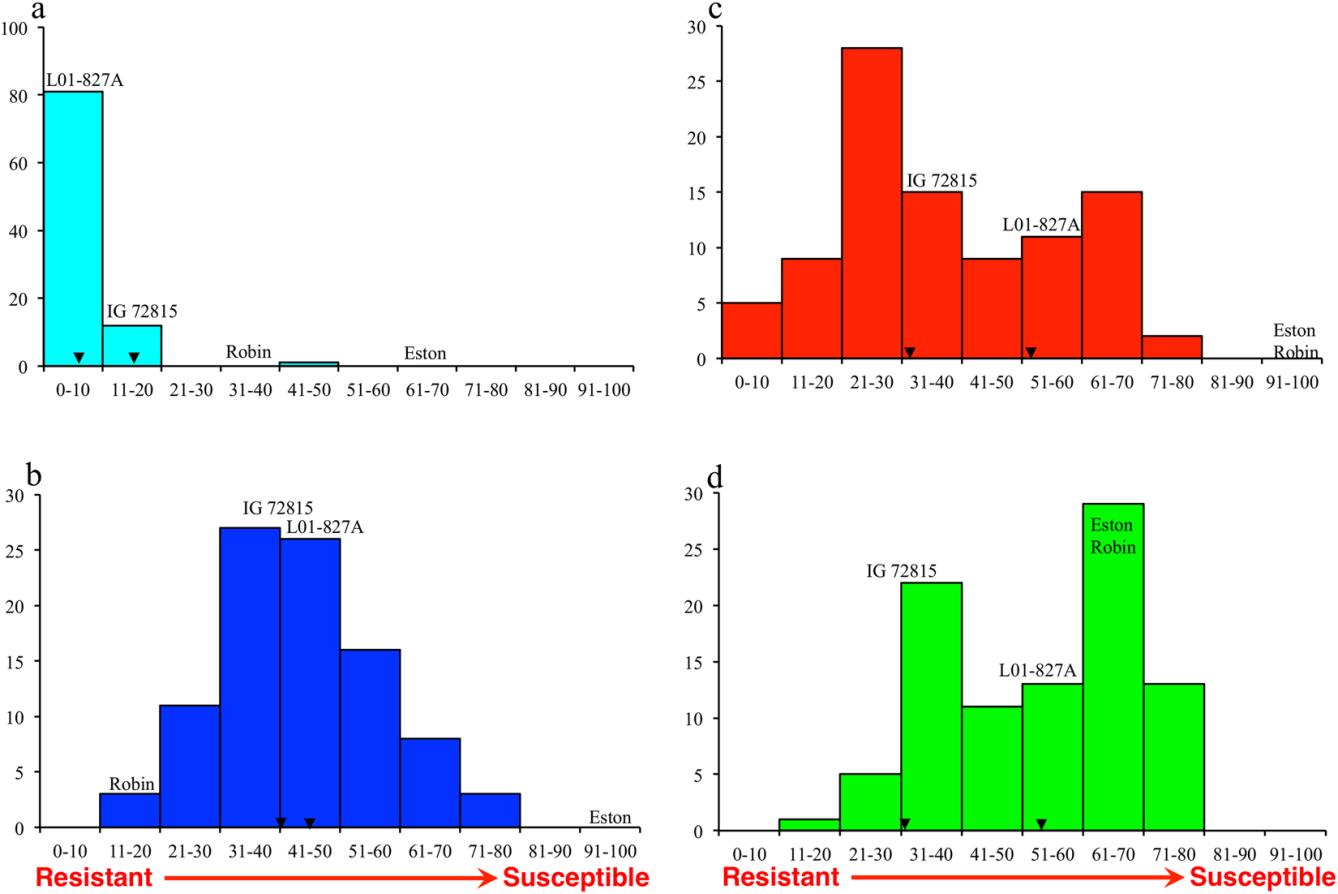
Frequency distributions of disease reactions in the *Lens ervoides* recombinant inbred line population LR-66 developed from L01–827A × IG 72815 (F_9_) following inoculation with *Ascochyta lentis* isolate AL61 (**a**), *Colletotrichum lentis* isolates CT-21 (race 1, **b**) and CT-30 (race 0, **c**), and *Stemphylium botryosum* isolate SB19 (**d**). Arrow heads indicate the least squares means of disease reactions on parental accessions. *Lens culinaris* cultivars Eston and CDC Robin are susceptible and resistant *L. culinaris* checks, respectively.

**Figure 2:**
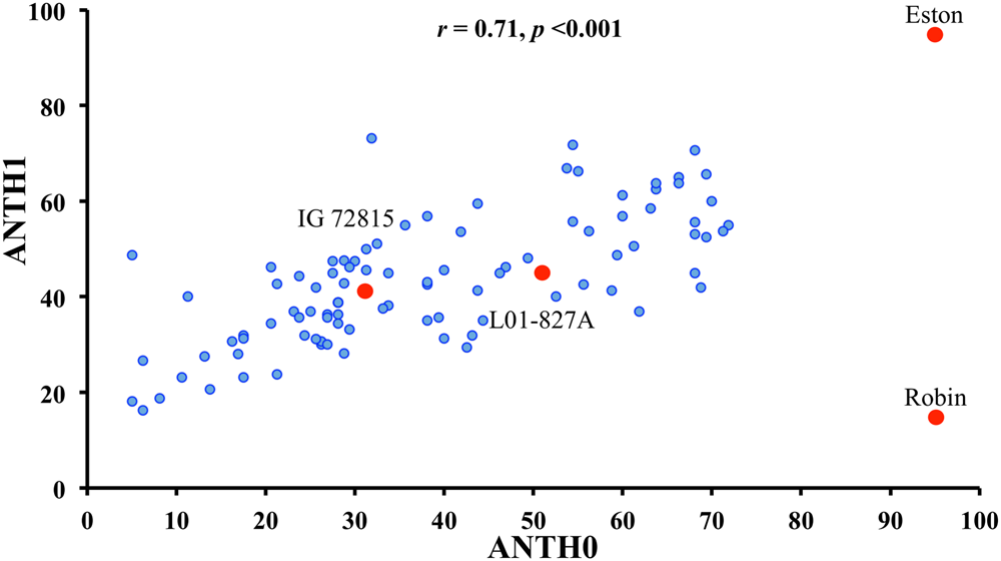
Correlation of disease reactions in the *Lens ervoides* recombinant inbred line population LR-66 developed from L01-827A × IG 72815 (F_9_) after inoculation with *Colletotrichum lentis* isolates CT-30 (race 0, X-axis) and CT-21 (race 1, Y-axis). r = Pearson’s correlation coefficient.

The accessions L01-827A and IG 72815 carry high to moderate levels of resistance to *C. lentis* race 0 (mean disease severity 51.9 and 31.9%), race 1 (45.0 and 40.3%), *A. lentis* (6.3% and 16.9%) and *S. botryosum* (53.1 and 30.6%). LR-66, an intraspecific RIL population previously developed from a cross between these two *L. ervoides* accessions was phenotyped for disease severity in response to *C. lentis*, *A. lentis and S. botryosum* inoculations in the greenhouse. Analysis of variance revealed significant variation in disease reactions among RILs to *C. lentis* races 0 and 1, and *S. botryosum* (*p* < 0.001). The RIL population did not segregate for resistance to *A. lentis* and therefore, those data were excluded from the further analysis (Fig. 1A). The frequency distribution of disease reactions to *C. lentis* race 1 revealed a monomodal and normal distribution (Shapiro-Wilk test, W = 0.99, *p* > 0.05) (Fig. 1B), suggesting polygenic inheritance of quantitative resistance in *L. ervoides*, whereas the responses to *C. lentis* race 0 (Fig. 1C) and to *S. botryosum* (Fig. 1D), appeared to follow bimodal distributions suggesting an oligogenic inheritance of resistance. Positive and negative transgressive segregations were detected in the LR-66 population for *C. lentis* and *S. botryosum* resistance, suggesting that alleles from both parental accessions L01-827A and IG 72815 contribute to the resistance. A significant positive correlation was observed between responses of RILs to *C. lentis* races 0 and 1 inoculations (Pearson’s correlation coefficient, *r = *0.71, *p* < 0.001), suggesting that some genes may regulate resistance to both races (Fig. 2).

### GBS of the LR-66 population, SNP discovery and segregation distortion

Sequencing of the 96-plex GBS library on the Illumina HiSeq 2500 platform yielded 289,072,291 100-bp paired-end reads, averaging 3,011,170 reads per RIL representing an average 7.53-fold genomic coverage for each RIL based on the estimated 4 Gb size of the *L. ervoides* genome. The reads were aligned against the *L. culinaris* ssp. *culinaris* cv. CDC Redberry genome assembly v0.8^22^, which resulted in 158,631,336 (54.88%) uniquely mapped, 107,406,271 (37.16%) multimapped reads and 23,034,683 (7.79%) unmapped reads. The uniquely mapped reads give a 4.13-fold genomic coverage to RILs with an average of 1,652,410 reads per RIL. A total of 422,885 single nucleotide polymorphisms (SNPs) between parental accessions L01-827A and IG 72815 were called from the uniquely mapped reads using the Bowtie2/SAMtools mpileup. Only 2,180 (0.52%) high-quality SNPs remained after a stringent post-mapping quality filtering based on an allele coverage ≥3-fold, <25% missing allele calls per locus and per RIL (SNP calls missing in 26 RILs or fewer), maximum 10% heterozygosity and 0.2–0.8 allele frequency. The SNPs were assembled into 543 unique haplotypes consisting of 368 genetic bins within which markers showed no evidence for recombination, and 175 singleton markers showing recombination with other markers (Table 1).

**Table 1:** Summary of the SNP-based linkage map of *Lens ervoides* L01-827A × IG 72815 RIL (F_9_) population.

Linkage group	Genetic bins	Markers in genetic bins	Singleton markers	Unique haplotypes	Genetic distance (cM)
LG1	50	339	23	73	105.98
LG2	67	301	20	87	129.28
LG3	64	303	36	100	119.07
LG4	50	312	26	76	95.27
LG5	47	227	26	73	86.59
LG6	48	252	27	75	107.91
LG7	42	271	17	59	96.84
Total	368	2005	175	543	740.94

The χ^2^ test revealed that 28% of the mapped SNP loci deviated significantly (*p* < 0.05) from the expected Mendelian 1:1 ratio of parental allele contribution. With an average of 1.07% instead of 0.39%, the LR-66 population showed a higher level of residual heterozygosity than expected for an F_9_ RIL population. The residual heterozygosity of individual RILs ranged from 0.14 to 11.21%.

### High-density genetic linkage map and comparative analysis

**Figure 3:**
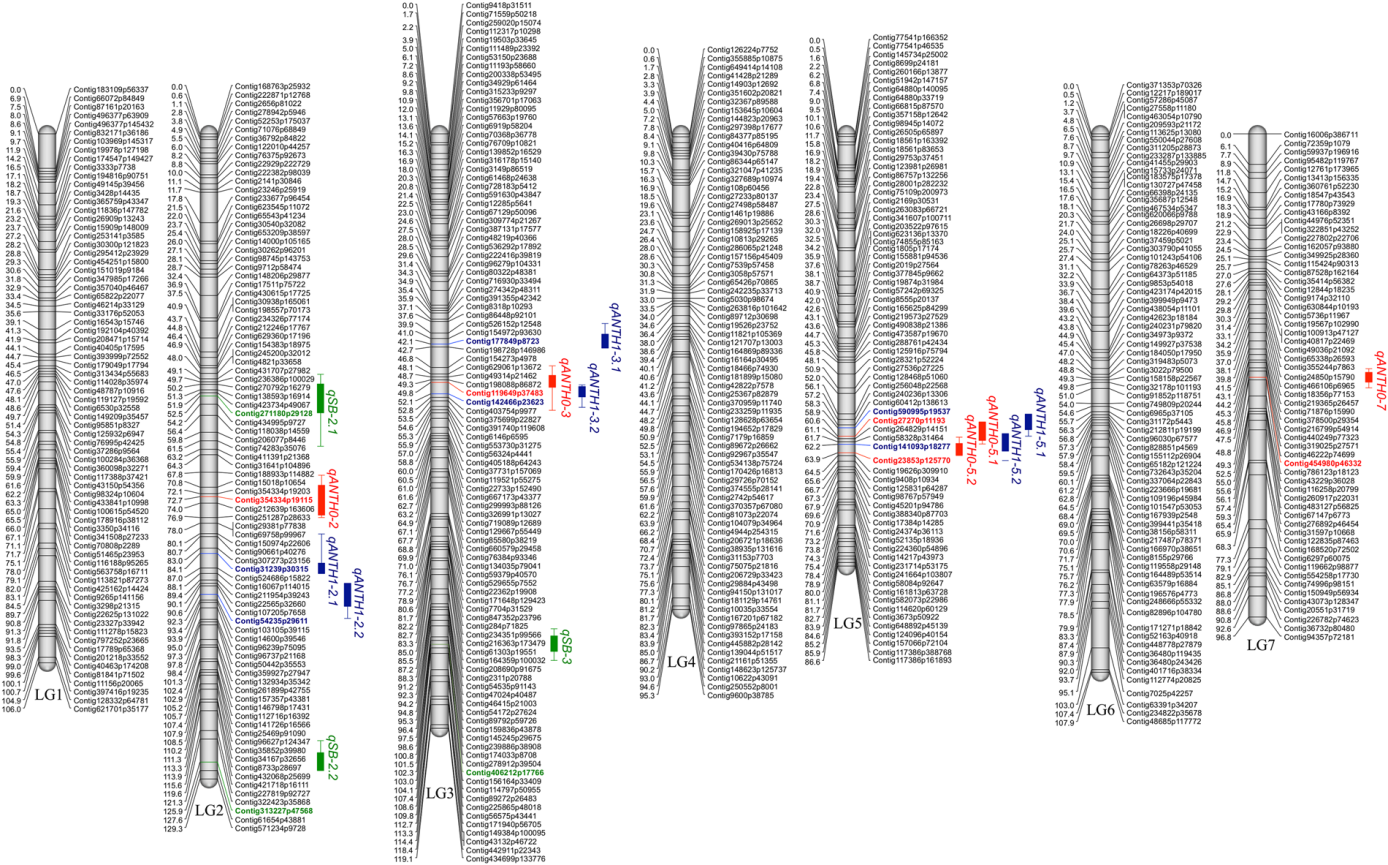
High-density genetic linkage map of the *Lens ervoides* recombinant inbred line population LR-66 (n = 94, F_9_) derived from L01-827A × IG 72815 and QTLs controlling resistance to three fungal pathogens. To the right of the linkage groups are SNP markers whose genetic positions in centimorgans are shown to the left of the linkage groups. The marker name indicates the position (p) of a SNP in the contig of the *Lens culinaris* genome assembly. QTLs conferring resistance to *Colletotrichum lentis* CT-30 (race 0) are shown as red, to *C. lentis* CT-21 (race 1) as blue, and to *Stemphylium botryosum* SB19 as green solid bars with 1-LOD confidence intervals. Vertical lines with caps on bars represent 2-LOD likelihood intervals.

**Figure 4:**
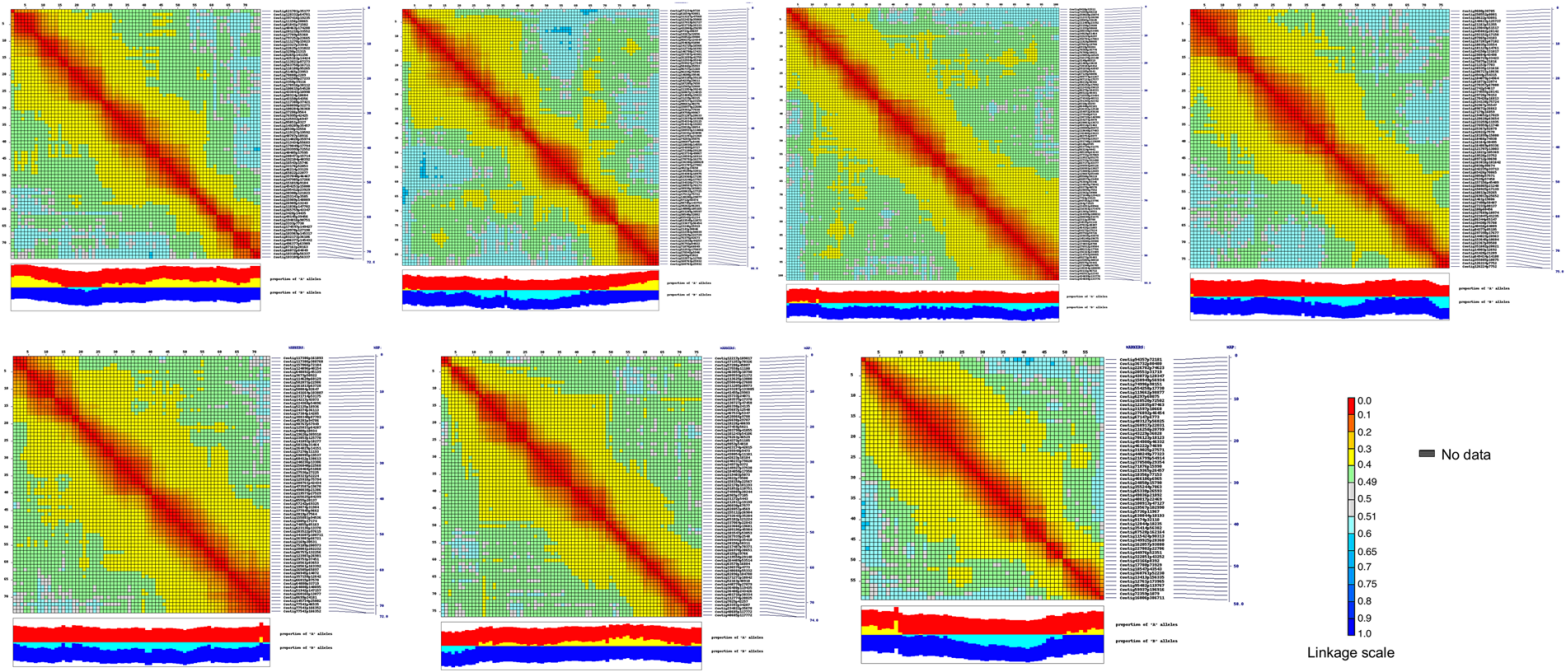
Validation of the genetic linkage map of *Lens ervoides* recombinant inbred line population LR-66. 2D-CheckMatrix heat plots were generated using the CheckMatrix python script. Each heat plot represents a linkage group. SNP markers are lined up against each other. A red block along the lines indicates tightly linked markers with low recombination. Panels beneath heat maps show the proportion of parental alleles (Red = L01-827A, Blue = IG 72815). Yellow (L01-827A) and light blue (IG 72815) colors in the middle represent segregation distortion.

Data from 543 SNP markers across 94 RILs were used to construct a linkage map. The SNP markers were mapped into 7 linkage groups (LGs, Fig. 3), which is equivalent to the haploid chromosome number in *L. ervoides*. CheckMatrix results confirmed the high-quality of the LR-66 genetic linkage map (Fig. 4), which spanned a cumulative distance of 741 cM with an average inter-marker distance of 1.36 cM. The number of SNP markers varied from 59 in LG7 to 100 in LG3, and the genetic distance from 86.59 cM in LG5 to 129.28 cM in LG2 (Table 1). The LGs were numbered to match the respective pseudomolecules of the *L. culinaris* genome^22^ as best as possible.

**Figure 5:**
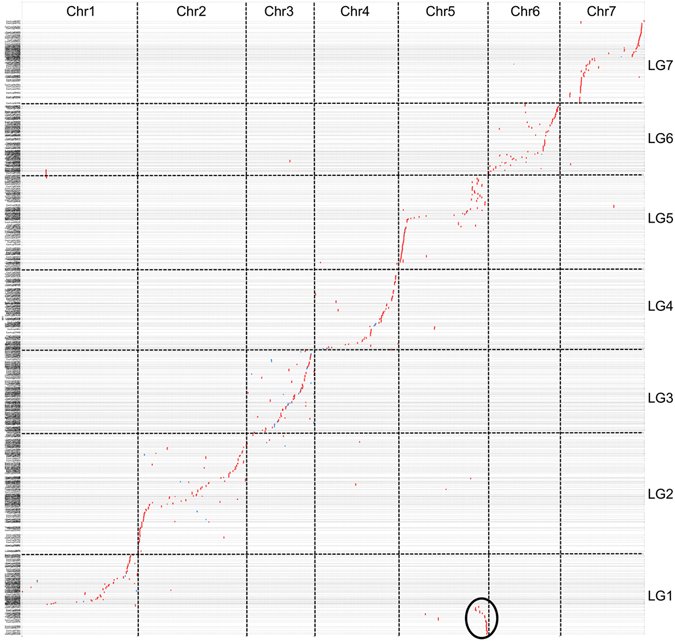
Dot plot representing collinearity between *Lens culinaris* pseudomolecules 1 through 7 (X-axis) and *L. ervoides* linkage groups 1 through 7 (Y-axis). A translocation between *L. culinaris* pseudomolecules 1 and 5 is circled.

In a previous study, a gene-based genetic map of *L. ervoides* was compared with the *L. culinaris* (Lc) LR-18 genetic map, suggesting a reciprocal translocation involving LcLG1 and LcLG5. However, due to an insufficient number of common markers, *Medicago truncatula* was used as an intermediate for comparative mapping^23^. In this study, we could directly compare the high-density genetic map of *L. ervoides* LR-66 with *L. culinaris* by mapping *L. ervoides* SNP loci onto the pseudomolecules of *L. culinaris*. This confirmed the chromosomal rearrangement in *L. culinaris* between chromosomes 1 and 5, providing a direct evidence for the presence of a reciprocal translocation (Fig. 5). The translocation was not evident when *L. ervoides* was compared to *M. truncatula* ^23^ so it must be unique to *L. culinaris*. Comparative analysis otherwise reveals a high level of collinearity between *L. ervoides* linkage groups and *L. culinaris* pseudomolecules (Fig. 5).

### Mapping of QTLs controlling resistance to anthracnose and Stemphylium blight

Composite interval mapping was performed using 543 SNPs and least squares means of disease reactions in percent. Five QTLs with a significant association with resistance to *C. lentis* race 0, six QTLs with association to *C. lentis* race 1 resistance, and three QTLs associated with *S. botryosum* resistance were identified in the LR-66 RIL population (LOD >3.0, *p* < 0.05) (Fig. 3, Table 2).

**Table 2:** QTLs controlling resistance to *Colletotrichum lentis* CT-30 (race 0, ANTH-0) and CT-21 (race 1, ANTH-1), *Stemphylium botryosum* (SB) in the *Lens ervoides* L01-827A × IG 72815 RIL population.

Trait	QTL	LG^1^	Peak marker	1-LOD^2^	2-LOD^2^	LOD	R^3^	ADD^4^	Allele source
ANTH-0	*qANTH0-2*	2	Contig354334p19115	70.4–76.4	68.4–76.9	5.00	10.94	−6.69	IG 72815
ANTH-0	*qANTH0–3*	3	Contig119649p37483	58.8–61.2	54.2–63.1	6.32	14.03	7.43	L01-827A
ANTH-0	*qANTH0-5.1*	5	Contig27270p11193	37.6–41.3	37.6–42	6.00	14.68	−7.60	IG 72815
ANTH-0	*qANTH0-5.2*	5	Contig23853p125770	31.1–33.5	29.8–33.5	8.05	18.82	−8.34	IG 72815
ANTH-0	*qANTH0-7*	7	Contig454980p46332	28.7–30.7	27.6–31.4	4.22	8.89	5.90	L01-827A
ANTH-1	*qANTH1-2.1*	2	Contig31239p30315	86–88.1	80.2–88.1	3.56	9.40	−4.34	IG 72815
ANTH-1	*qANTH1-2.2*	2	Contig54235p29611	90.1–94.7	90.1–97.1	4.08	10.00	−4.24	IG 72815
ANTH-1	*qANTH1-3.1*	3	Contig177849p8723	64.9–67.7	64.9–69.8	4.20	14.22	5.02	L01-827A
ANTH-1	*qANTH1-3.2*	3	Contig142466p23623	57.6–59.7	55.5–60	8.67	24.75	6.72	L01-827A
ANTH-1	*qANTH1-5.1*	5	Contig590995p19537	38.7–41.8	38.7–43.1	5.54	14.68	−5.19	IG 72815
ANTH-1	*qANTH1-5.2*	5	Contig141093p18277	32.4–35.9	34.8–35.9	5.64	15.39	−5.24	IG 72815
SB	*qSB-2.1*	2	Contig271180p29128	50.1–55.9	48.2–62.6	3.40	9.91	−5.22	IG 72815
SB	*qSB-2.2*	2	Contig313227p47568	124–127.6	121.6–127.6	5.82	18.29	6.80	L01-827A
SB	*qSB-3*	3	Contig406212p17766	9.1–12.2	7.3–13.6	4.11	12.30	−5.75	IG 72815

^1^LG, Linkage group.

^2^1-LOD and 2-LOD support interval.

^3^R^2^, phenotypic variance explained in per cent.

^4^ADD, Additive genetic effect. Positive and negative signs indicate that alleles for the corresponding traits are derived from the parental accessions L01-827A and IG 72815, respectively.

Three QTLs (*qANTH0-3*, *qANTH0-5.1* and *qANTH0-5.2*) accounting for 47.58% of the variability in resistance to *C. lentis* race 0 were co-localized within the 2-LOD support interval of three QTLs (*qANTH1-3.2*, *qANTH1-5.1* and *qANTH1-5.2*) accounting for 54.82% of the variability to race 1 resistance in *L. ervoides*. L01-827A and IG 72815 contributed one (LG3, additive effect +7.43 [race 0] and +6.72 [race 1]) and two (LG5, additive effect −8.34 and −7.60 [race 0], and −5.24 and −5.19 [race 1]) positive alleles for anthracnose resistance, respectively. The remaining two *C. lentis* race 0 resistance QTLs *qANTH0-2* (LOD 5.0) and *qANTH0-7* (LOD 4.22) were mapped on LG2 and LG7, and accounted for 10.94 and 8.89% of the phenotypic variance among RILs, respectively. The remaining three QTLs for *C. lentis* race 1 resistance - two on LG2 (*qANTH1-2.1* and *qANTH1-2.2*) and one on LG3 (*qANTH1-3.1*), had LOD scores 3.56, 4.08 and 4.20, respectively, and together explained 33.62% of the phenotypic variance among RILs. The QTLs for *S. botryosum* resistance were located on LG2 (*qSB-2.1* and *qSB-2.2*) and LG3 (*qSB-3*), and together contributed to 40.5% of the phenotypic variance among RILs. Similar to *C. lentis* resistance, both parental accessions contributed positive alleles (one L01-827A and two IG 72815 alleles) to *S. botryosum* resistance (Table 2). Differing proportions of variation − 32.64% for *C. lentis* race 0, 11.56% for *C. lentis* race 1 and 59.5% for *S. botryosum*, remained unmapped.

## Discussion

Lentil has a narrow genetic base presumably linked to a bottleneck created at the time of domestication when it underwent unconscious selection for a small number of traits, such as shatter-resistant seed pods, larger seed size and lack of secondary dormancy^24, 25^. As a result, in the absence of sufficient genetic diversity, the co-evolutionary arms race allowed pathogens not only to overcome basal resistance (leading to enhanced susceptibility), a key component of quantitative resistance^26^, but also to defeat effector-triggered immunity (leading to a collapse of resistance) either through mutation (direct interactions) or jettison (indirect interactions) of pathogen effectors under selection pressure. This has necessitated a renewed broadening of the genetic base of the cultivated genepool for sustainable lentil production. Wild relatives of lentil, especially *L. ervoides* in the tertiary genepool, have been identified as rich source of genetic diversity that can be exploited to improve agronomic traits, such as disease resistance^15^. Interspecific introgression of new genes of interest into cultivated lentil using marker-assisted selection could provide a natural way to incorporate resistance at an accelerated speed, but segregation distortion in interspecific populations has prevented the development of such markers^27^. In this study, we applied quantitative genetics approach to map allelic diversity controlling resistance to fungal pathogens in an intraspecific population of *L. ervoides*.

*Lens ervoides* accessions L01-827A and IG 72815 show varying levels of resistance to *C. lentis*, *A. lentis* and *S. botryosum* (Fig. 1) that are expected to be conferred by resistance genes/alleles not found in *L. culinaris*. It has been hypothesized that one of the key components of quantitative resistance is basal defense where perception of conserved pathogen molecules by transmembrane receptors triggers partial (quantitative basal resistance) or complete (qualitative basal resistance or non-host resistance) defense. Effector-triggered immunity likely contributes to the basal resistance^28^. For example, recognition of chitin oligosaccharides by LysM RLK1 elicits focal induction of defense-related proteins, such as chitinases that leads to quantitative resistance in *Arabidopsis thaliana* against fungal pathogens, such as *Erysiphe cichoracearum* and *Alternaria brassicicola* whereas mutation in *LysM RLK1* leads to enhanced susceptibility^29^. The presence of transgressive resistant offspring, such as LR66-528 (*C. lentis* race 0 resistance) and LR66-637 (*S. botryosum* resistance) (Fig. 1) suggests that positive alleles were contributed by both parents, and that rearrangement or complementation of parental alleles may be reinforcing basal resistance in transgressive RILs. GBS allows for such alleles to be tracked at a high resolution in segregating populations. Using *Ape*KI-based GBS, Felderhoff *et al*. mapped two loci (*qCs-7* and *qCs-9*) regulating resistance to *C. sublineola* in a biparental segregating population of sorghum (*Sorghum bicolor*) on a high-density (5,186 SNP markers) linkage map^30^. These loci span 48.7 Mb (*qCs-7*) and 4.5 Mb (*qCs-7*), having 40 and 36 genes implicated in disease resistance, including NBS-LRR class of resistance proteins, chitinases, defensins, peroxidases, polyphenol oxidases, germin like proteins and ABC transporters.

Genotyping of LR-66 using *Pst*I and *Msp*I restriction enzymes to fragment the genome prior to sequencing resulted in the discovery of 2,180 SNP markers distributed across 7 linkage groups with a cumulative spanning distance of 741 cM (Fig. 3). QTL analysis identified 5 QTLs controlling resistance to *C. lentis* race 0 and 6 QTLs for race 1 resistance, and 3 QTLs for *S. botryosum* resistance. At nine of the fourteen QTLs, the resistance allele was contributed by IG 72815 whereas L01-827A contributed the resistance allele at the remaining 5 QTLs. This suggests that the rearrangement of alleles from both parents is required to trigger the highest level of resistance response to the pathogens (Table 2). The QTL for resistance to *C. lentis* races 1 and 0 co-localized on LG3 (*qANTH0-3* and *qANTH1-3.1*) and LG5 (*qANTH0-5.1* and *qANTH0-5.2*; *qANTH1-5.1* and *qANTH1-5.2*), collectively accounting for 47.58% and 54.82% of the variance in resistance response to *C. lentis* races 0 and 1, respectively. This suggests that a large proportion of the resistance in LR-66 to the two races of *C. lentis* is regulated by genes at the same loci, which is consistent with the positive correlation between the two traits (*r = *0.71, *p* < 0.001) (Fig. 2). Differential responses of RILs to races 0 and 1 of *C. lentis* are likely associated with 2 unique loci (*qANTH0-2* and *qANTH0-7*) explaining the remaining 19.83% of the variance in race 0 resistance, and 3 unique loci (*qANTH1.2-1*, *ANTH1.2-2* and *qANTH1.3-2*) explaining 33.62% of the variance in race 1 resistance. QTL analysis identified 3 loci conferring resistance to *S. botryosum*, 2 on LG2 (*qSB-2.1* and *qSB-2.2*) and 1 (*qSB-3*) on LG3 with resistance alleles derived from both parents. Collectively, they account for 40.5% of the variance in the LR-66 population. The unmapped genetic variation in all cases is likely conferred by genes with minor effects, falling below the statistical threshold of detecting QTLs.

Comparative mapping between the high-density genetic map of *L. ervoides* LR-66 with the *L. culinaris* chromosomes provides new insights into the genome evolution of cultivated lentil as a reciprocal translocation between chromosomes 1 and 5 was identified in the *L. culinaris* genome. A high level of collinearity between the two genomes, especially in the identified QTL regions suggests that in the absence of a *L. ervoides* genome, *L. culinaris* can be utilized to identify homologous candidate genes for traits of interest, such as disease resistance (Fig. 5). It is also possible to deploy disease resistance QTLs in *L. culinaris* via interspecific hybridization with *L. ervoides* and selection using the SNP markers linked to these QTLs.

## Methods

### Plant material and fungal isolates

The bi-parental RIL population LR-66 was developed from a single F1 plant derived from a cross between *L. ervoides* accessions L01-827A and IG 72815^23^ followed by single seed descent from the F2 population and 94 RILs, bulked at the F_9_ generation, were genotyped and phenotyped. *Lens culinaris* cultivars Eston^31^ and CDC Robin^32^ were used as controls for successful inoculations.

*Colletotrichum lentis* isolates CT-30 (highly virulent race 0) and CT-21 (less virulent race 1), *A. lentis* isolate AL-61, and S*. botryosum* isolate SB-19, all of which are field isolates from commercial lentil fields in the Canadian Province of Saskatchewan, were routinely maintained on oatmeal or oatmeal V8 agar plates supplemented with 0.01% chloramphenicol^33^.

### Phenotyping of LR-66 for disease reactions

Parental accessions L01-827A and IG 72815, 94 RILs and Eston and CDC Robin were planted in the greenhouse. The experimental design was a 4 × 98 factorial arranged in a randomized complete block design with 4 replicates, which were blocked over time. The factors were pathogen isolates *C. lentis* CT-21 (race 1), *C. lentis* CT-30 (race 0), *A. lentis* AL-61 and *S. botryosum* SB19, and 98 lentil genotypes. Four plants per genotype were grown in 4 inch square pot filled with SUNSHINE professional growing mix 4 (Sun Gro Horticulture, Bellevue, USA) and perlite (Special Vermiculite Canada, Winnipeg, Canada) in 3 to 1 ratio, and 4 replicate pots were prepared per isolate/genotype combination. Twenty five-day old plants were spray-inoculated with isolate AL-61 at a concentration of 5 × 10^5^ conidia ml^−1^, CT-30 and CT-21 isolates at 5 × 10^4^ conidia ml^−1^, or SB-19 isolate at 1 × 10^5^ conidia ml^−1^ at approximately 3 ml of conidial suspension per plant depending on the experiment. Plants were incubated in high humidity for 48 hours in the case of AL-61 and SB-19, and for 24 hours in the case of CT-30 and CT-21, before being moved to a greenhouse bench with 30 seconds misting every 90 minutes. Individual plants were scored for *C. lentis* and *A. lentis* severity 7 and 21 days post-inoculation (dpi), respectively, using a 0 to 10 rating scale with 10% increments in disease severity. Severity of *S. botryosum* infection was assessed with a semi-quantitative rating scale 7 dpi where 0: healthy plants; 1, few tiny lesions; 2, a few chlorotic lesions; 3, expanding lesions on leaves, onset of leaf drop; 4, 1/5th of nodes affected by lesions and leaf drop; 5, 2/5th of nodes affected; 6, 3/5th of nodes affected; 7, 4/5th of nodes affected; 8, all leaves dried up; 9, all leaves dried up but stem green; and 10, plant completely dead.

Disease scores were averaged per replicate pot and subjected to analysis of variance using the mixed model procedure of SAS v.9.3 (SAS Institute, Cary, USA). The presence of transgressive segregants was assessed with the LSMEANS statement. Shapiro-Wilk test implemented in PROC UNIVARIATE (SAS v9.3) and mixtools implemented in R were used to determine the distribution of disease severity in case of monomodal distribution.

### GBS library construction and Illumina sequencing

Genomic DNA (gDNA) was extracted from pooled leaf tissues of the parental accessions L01-827A and IG 72815 and 94 RILs using the DNeasy Plant Mini Kit (Qigen, Inc., Hilden, Germany). A GBS library was constructed following the protocol developed by Poland *et al*.^21^. In brief, two hundred ng gDNA per RIL was digested with the 6-base cutter *Pst*I (CTGCAG) and the 4-base cutter *Msp*I (CCGG) in an Eppendorf^®^ 96-well twin.tec PCR plate (Eppendorf, Hamburg, Germany). Forward adapters with 4–8 bp barcodes with *Pst*I overhang (TGCA) and a common reverse Y-adapter with *Msp*I overhang (CG) were ligated to restriction digests using T4 DNA ligase (New England Biolabs, Inc., Ipswich, USA). The 96-plexed library was then amplified using the Illumina paired-end primer set (IlluminaF_PE/IlluminaR_PE). The Y-adapter eliminates the amplification of *Pst*I/*Pst*I and *Msp*I/*Msp*I digests, hence yields *Pst*I/*Msp*I fragments. The sequence-ready GBS library was checked for primer dimer and adapter dimer contaminations. Four serial dilutions derived from 1 µl of the library were subjected to the Agilent 2100 Bioanalyzer (Agilent Technologies, Inc., Palo Alto, USA) analysis using the Agilent 1000 DNA chip. The GBS library was quantified using the KAPA library quantification kit for Illumina sequencing platforms (KAPA Biosystems, Inc., Woburn, USA). The 20 pM 96-plex library free of primer, adapter and primer-adapter dimer contamination was sequenced on the Illumina HiSeq 2500 platform (PE 2 × 100 bp; Illumina, Inc., San Diego, USA) at The Centre for Applied Genomics, The Hospital for Sick Children, Toronto, Canada, or Le Centre d’Innovation Génome Québec, Montréal, Canada.

### Variant calling and post-mapping quality filtering

Paired-end sequencing data were de-multiplexed according to their barcodes and assigned to respective RILs using a custom Perl script. Reads with *Pst*I and *Msp*I restriction sites were clipped using Trimmomatic^34^ with a sliding window size of 4 bases and an average base quality threshold of 30. Remaining reads were then mapped onto the *L. culinaris* spp. *culinaris* cv. CDC Redberry v0.8 genome assembly using the end-to-end mode of Bowtie 2^35^. SNP variants were called where parental accessions showed polymorphism, and were recorded in the sequence alignment and mapping format, then converted to binary format (BAM). In total, 96 variant call format (VCF) files were generated from BAM files using SAMtools mpileup^36^. The files were then merged into a population VCF file prior to linkage analysis of SNP markers.

The SNPs were scanned for allele depth using vcffilter, and allele calls with two or fewer read support were converted to missing values. vcftools was employed to remove markers and genotypes with 25% missing allele calls, and markers with minor allele frequency of less than 0.33 were also removed. A maximum of 10% heterozygosity per marker was allowed. SNPs were named based on the *L. culinaris* contig number followed by the position (p) of the base.

### Genetic linkage mapping, QTL detection and comparative mapping

High-quality SNP markers were clustered into linkage groups using the MadMapper python script^37^ with the following setting: a recombination fraction cut-off of 0.2, a BIT score cut-off of 100, a data cut-off of 25 (minimum number of markers in a linkage group), missing data of 25 (missing allele calls), minor allele frequency of 0.33 and NOTRIO (no triplet analysis). The RECORD algorithm implemented in the Windows version of RECORD^38^ was employed to determine the linear order of markers within the linkage groups using the following setting: recombination fraction of 0.1, 30 cM gap size and Kosambi mapping function. COUNT rippling algorithm with a window of 5 markers was used to fine-tune the linear order of markers.

Individual linkage groups were visualized using the CheckMatrix python script^37^ and the color genotype functionality of MapDisto^39^ for potential genotypic errors, such as double recombinants. After error correction, the linear order of markers within the linkage groups was re-determined using RECORD algorithm^38^. The marker order within a linkage group that spans the shortest genetic distance was chosen as the final linkage group.

To validate the linkage map, a 2D-CheckMatrix heat map was plotted for each linkage group using the CheckMatrix python script^37^ wherein SNP markers were lined up against each other to evaluate marker order and mapping quality. Red (recombination fraction <0.2) diagonal lines were generated and buffered along the entire length by a yellow zone (recombination fraction 0.2–0.4), indicating that the markers were assigned to correct linkage groups in the correct order. Red blocks along the diagonal lines represent tightly linked markers with low recombination (Fig. 4).

Composite interval mapping (CIM) with the standard model 6 (Zmapqtl 6) implemented in the Windows version of the QTL Cartographer v2.5^40^ was used to map QTLs onto the genetic linkage map. QTL thresholds were determined by a permutation test with 1000 iterations at a significance level of 0.05. For controlling the genetic background, a set of markers (cofactors) across the map was selected using forward and backward stepwise regression. CIM was performed with a walking speed of 1 cM, a window size of 10 cM and a probability of into or out of 0.1.

Homologous sequences between *L. ervoides* and *L. culinaris* were identified using NUCmer and visualized using MUMmerplot of the MUMmer software^41^.
